# *Mi PROTECT*: A personalized smartphone platform to report back results to participants of a maternal-child and environmental health research cohort program in Puerto Rico

**DOI:** 10.1371/journal.pdig.0000172

**Published:** 2023-01-18

**Authors:** Nancy R. Cardona Cordero, Irene Lafarga Previdi, Héctor R. Torres, Ishwara Ayala, Katherine E. Boronow, Amailie Santos Rivera, John D. Meeker, Akram Alshawabkeh, José F. Cordero, Julia Green Brody, Phil Brown, Carmen M. Vélez Vega

**Affiliations:** 1 Department of Social Sciences, School of Public Health, Medical Sciences Campus, University of Puerto Rico, San Juan, Puerto Rico; 2 Center for Collaborative Research in Health Disparities, Medical Sciences Campus, University of Puerto Rico, San Juan, Puerto Rico; 3 College of Engineering, Northeastern University, Boston, Massachusetts, United States of America; 4 Silent Spring Institute, Newton, Massachusetts, United States of America; 5 Department of Environmental Health Sciences, School of Public Health, University of Michigan, Ann Arbor, Michigan, United States of America; 6 Department of Epidemiology and Biostatistics at the University of Georgia’s College of Public Health, Athens, Georgia, United States of America; 7 Social Science Environmental Health Research Institute, Northeastern University, Boston, Massachusetts, United States of America; Tsinghua University, CHINA

## Abstract

**Background:**

The PROTECT Center is a multi-project initiative that studies the relationship between exposure to environmental contaminants and preterm births during the prenatal and postnatal period among women living in Puerto Rico. PROTECT’s Community Engagement Core and Research Translation Coordinator (CEC/RTC) play a key role in building trust and capacity by approaching the cohort as an engaged community that provides feedback about processes, including how personalized results of their exposure to chemicals should be reported back. The goal of the *Mi PROTECT* platform was to create a mobile-based application of DERBI (Digital Exposure Report-Back Interface) for our cohort that provides tailored, culturally appropriate information about individual contaminant exposures as well as education on chemical substances and approaches to exposure reduction.

**Methods:**

Participants (N = 61) were presented with commonly used terms in environmental health research related to collected samples and biomarkers, followed by a guided training on accessing and exploring the *Mi PROTECT* platform. Participants evaluated the guided training and *Mi PROTECT* platform answering a Likert scale in separated surveys that included 13 and 8 questions, respectively.

**Results:**

Participants provided overwhelmingly positive feedback on the clarity and fluency of presenters in the report-back training. Most participants reported that the mobile phone platform was both accessible and easy to navigate (83% and 80%, respectively) and that images included in the platform facilitated comprehension of the information. Overall, most participants (83%) reported that language, images, and examples in *Mi PROTECT* strongly represented them as Puerto Ricans.

**Conclusions:**

Findings from the *Mi PROTECT* pilot test informed investigators, community partners and stakeholders by demonstrating a new way to promote stakeholder participation and foster the “research right-to-know.”

## Author summary

The PROTECT Center focuses on studying how contaminants in the environment can affect pregnancy outcomes in women living in the northern Karst of Puerto Rico. In order to facilitate reporting back research results to study participants, the research team decided to develop a mobile based application called Mi PROTECT. Before sharing this application with all study participants, some participants were consulted to ensure that the content was understandable, accessible and culturally appropriate. This was done through in-person meetings where they learned how to navigate the application and revised the content (personal results, definition of contaminants, recommendations to reduce exposure) and provided feedback through an evaluation survey. Results from the survey indicate that participants provided overwhelmingly positive feedback regarding the use of Mi PROTECT. This is an important initiative that demonstrates the relevance of the research right to know and that research participants should be consulted and considered when designing strategies to report back study findings.

## Introduction

There is a growing consensus in the biomedical literature that disclosing research results to study participants is ethical, offers benefits to participants, and can be accomplished without harm [1]. A report-back strategy provides study participants with the knowledge of potential risks and harms of personal exposures in order to make more informed decisions [2]. Report-back can be conducted in a variety of modes (i.e., in-person, telephone, mail, web application, smartphone) either individually or in a group/community setting and is a valuable asset to enhance participants’ health literacy and overall engagement, especially when the initiative is culturally-aware. Ramírez-Andreotta and colleagues [3] found that participants of the *Metals Exposure Study in Homes* used the information received to change their families’ household behaviors and initiated specific interventions to reduce family exposures. In the Northern California Household Exposure Study, the combination of individual and community report-back engaged participants in a deeper and more collaborative way, thereby giving them an opportunity to analyze their data, exchange ideas on solutions, and take collective action [4]. Report-back includes approaches that enhance environmental health literacy, encouraging a broad array of science-based practices to develop skills and competencies that promote informed individual choices as well as social and environmental interventions at the community level [3,5–6]. The practice of reporting back not only benefits participants but also researchers, as shown in the Household Exposure Study [7].

**Fig 1:**
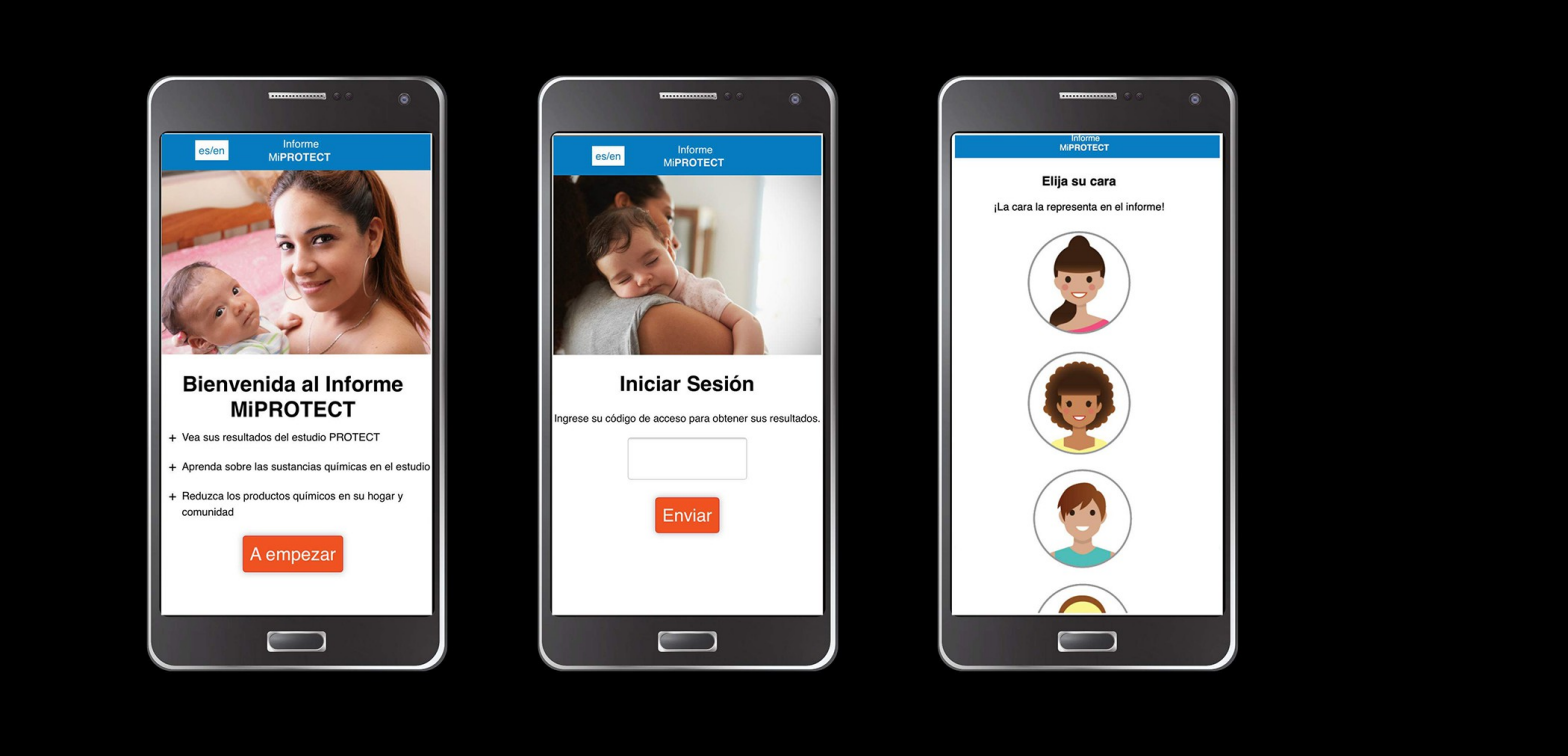
*Mi PROTECT* mobile platform access and avatar selection.

Digital technology is needed to facilitate reporting. The Silent Spring Institute developed the Digital Exposure Report-Back Interface (DERBI) as a digital method that promotes “right-to-know” by allowing the timely sharing of personal exposure results with study participants [8]. DERBI produces personalized summaries of results and generates graphs of individual results with comparisons to the study group and to benchmarks from the National Health and Nutrition Examination Survey (NHANES). Participants receive unique ID codes to access their individualized report and have the option of selecting an avatar with their resemblance (Fig 1). The reports include potential sources of chemical exposure, possible related health effects, tactics to reduce exposure and general findings from the project. As such, DERBI has dual potential for advancing the sociological importance of report-back and scientific analysis of multiple exposures while also providing a printed or computer-based and mobile report-back for participants [9].

Demonstrating that this tool can be adapted to diverse populations is an urgent need because of the increased burden of pollution among communities of color and low socioeconomic levels. The PROTECT Center, a Superfund Research Program funded by the National Institutes of Health (NIH), offered a unique opportunity to adapt and evaluate DERBI in a LatinX (Latin American cultural or ethnic identity) population of pregnant people residing in a highly contaminated environmental area with a high burden of adverse reproductive outcomes [10].

**Fig 2:**
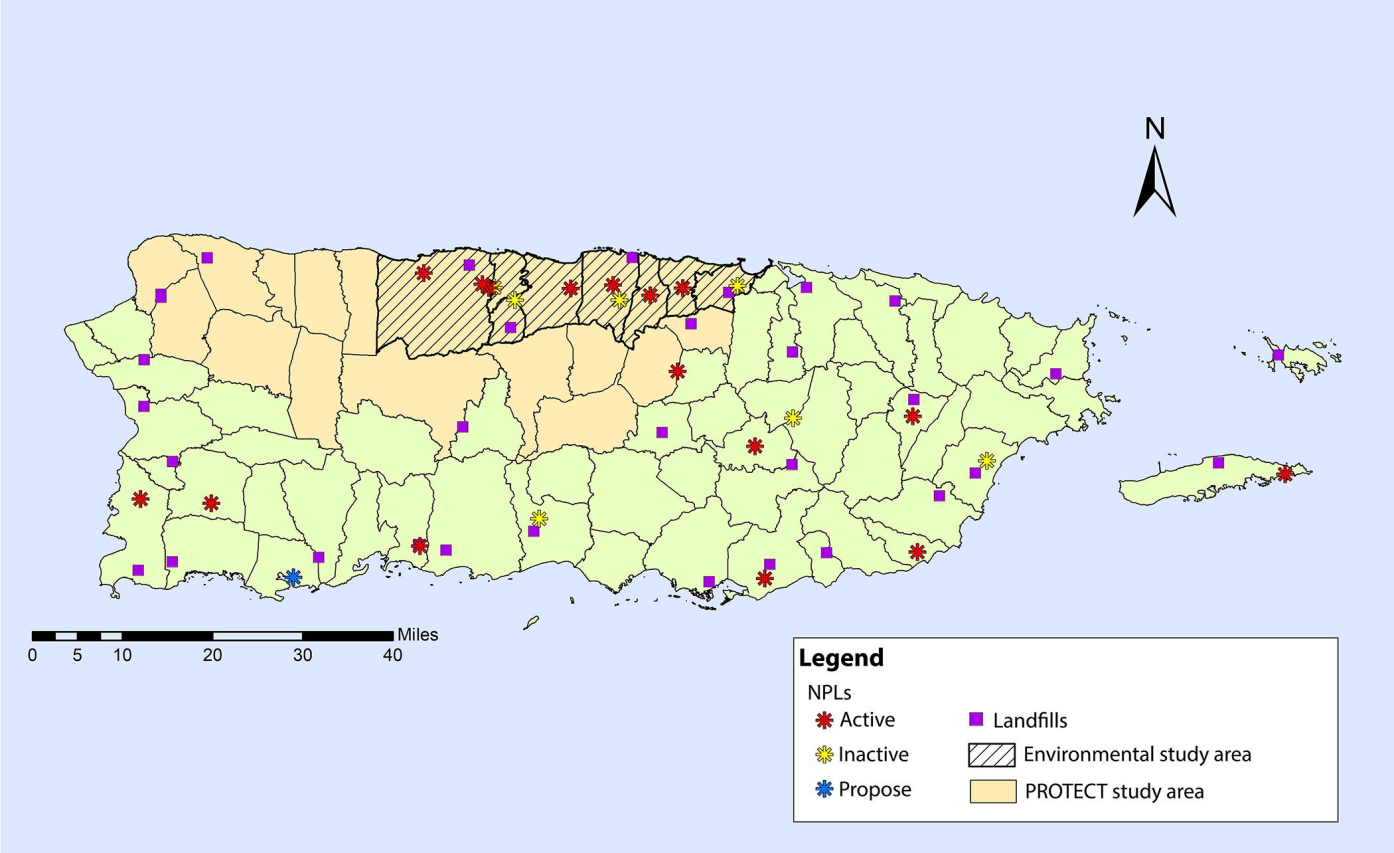
Map of Puerto Rico National Priority List of Superfund Sites and research area covered by the PROTECT Center.

The PROTECT Center examines the role of persistent organic pollutants (POPs) on adverse reproductive outcomes, particularly preterm births (gestation <37 weeks). Puerto Rico has a history of a high rate of preterm births, reaching nearly 20% in 2008, and also has a high level of contaminated sites with the highest density per square mile of polluted sites in the U.S. Environmental Protection Agency (EPA) National Priority List (Fig 2). PROTECT’s community engagement approach merges concerns for environmental justice with reproductive health [11] and the research right-to-know principles described by Morello-Frosch et al. [12]. Puerto Rico’s history of unethical procedures and methods impacting women’s health [13–14] renders this effort even more compelling.

The PROTECT Center was developed with a community-based participatory research framework driven by community-based organizations, including the March of Dimes Foundation and local environmental groups such as Ciudadanos del Karso (Citizens of the Karst), Basura Cero (Zero Waste), Comité Aceitunas y Arenales Unidos por la Salud y el Ambiente (Aceitunas and Arenales Committee United for Health and the Environment), and others. We report on the development and evaluation of a DERBI-adapted tool in a Puerto Rican population of pregnant people residing in a highly polluted area.

Our aims were (1) to pilot a DERBI interface adapted to our LatinX population and (2) evaluate among PROTECT participants the acceptance, relevancy, and cultural appropriateness of the tool, including avatars, the graphical representation of reported data and the channels to deliver the individual report-back information. Our overall goal was to return results through an innovative, culturally appropriate approach that addresses PROTECT participants’ expectations for report-back. We hypothesize that participants will consider the DERBI *Mi PROTECT* as a tool that provides easy-to-understand and relevant information that is culturally appropriate.

## Methods

### Study setting

This study occurs in the PROTECT study area: the Northern Karst region of Puerto Rico that contains 10 of the 22 EPA Superfund hazardous waste sites on the island [15] (see Fig 2). Risk of Superfund contaminants reaching the groundwater (and ultimately tap water) is high in Puerto Rico as many of these sites are unlined landfills that overlie karst aquifers, which are highly permeable pathways for exposure to contamination [16].

### *Mi PROTECT*: Development of the report-back platform

**Fig 3:**
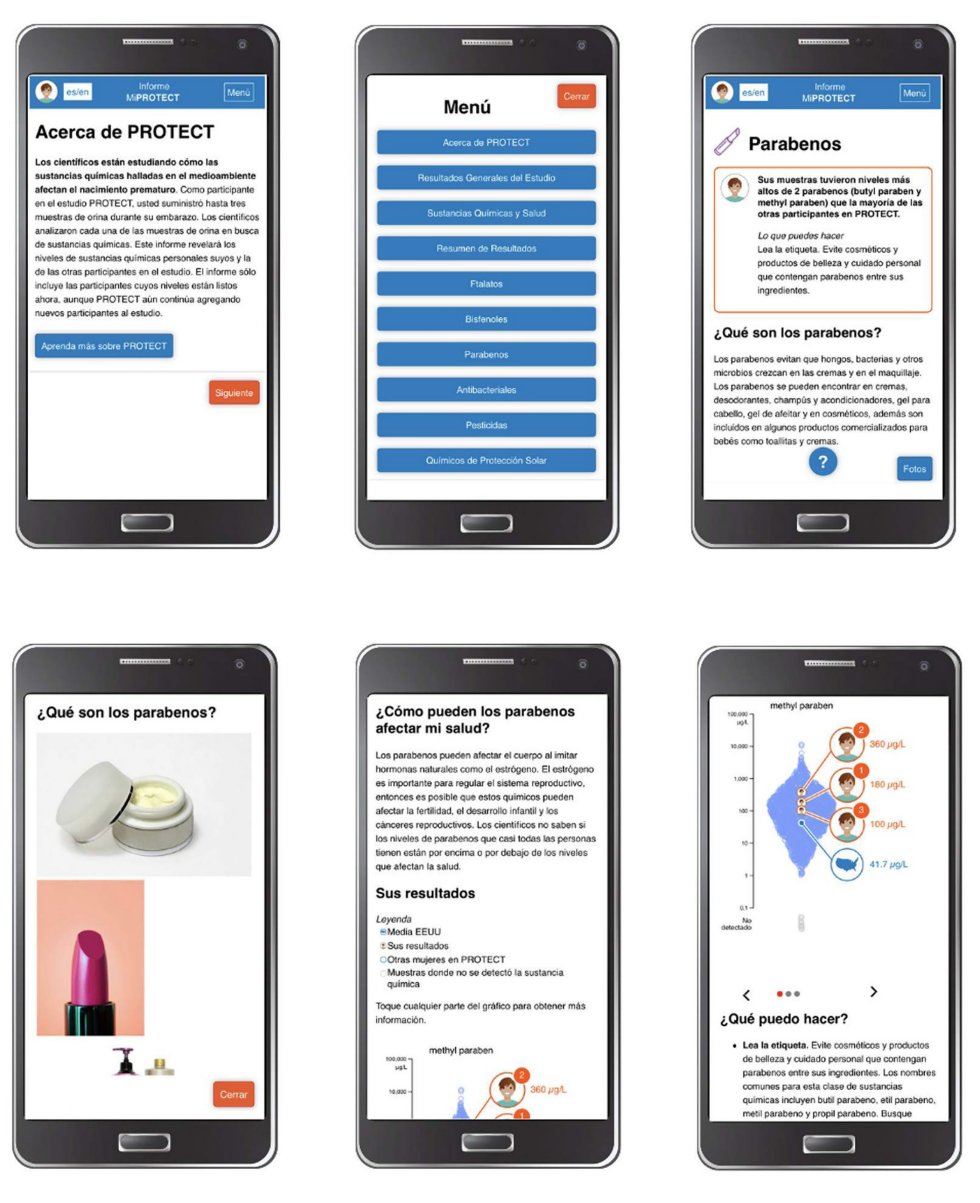
View of general information about PROTECT and personalized results section. a) General overview of the PROTECT program. b) Menu section with options to access general results and personalized results by substance. c) Brief overview of personalized results and recommendations on how to reduce exposure are shown inside the orange square at the top part of the screen. Detailed information about the selected substance and access to pictures of products that may contain this substance are at the bottom part of the screen. d) Pictures of consumer products that may contain this substance and a close option to return to the previous screen. e) Detailed information about how selected substances may affect your health and legend of graphs that provide results. f) Graph of the three (3) biological samples taken during pregnancy and mean level of this substance in the U.S. population based on NHANES Study.

The DERBI platform developed by Silent Spring Institute was adapted for the PROTECT study, within the framework of describing the chemicals that were found, the potential health concerns, what participants could do, health concerns, and overall study results. The adapted tool “*Mi PROTECT*” (Spanish for “My PROTECT”) includes 1) general introductory information on the PROTECT Center; 2) urine levels of up to six (6) environmental chemicals, by visit; 3) levels of environmental chemicals in household tap water, if collected; 4) recommendations on how to reduce exposure to each chemical found and 5) overall study results summarizing what has been learned from the study as a whole. Chemical results, and associated contextual information about sources and health effects, are divided into groups of related chemicals (Bisphenols, Antibacterials, Parabens, Pesticides, Phthalates, Sunscreen chemicals and CVOCs). Information is specified with text and graphs and includes comparison with both the study population and to similarly aged U.S. women tested in NHANES (Fig 3). *Mi PROTECT* is bilingual and the participant can choose to use the application in Spanish or English. Participants can navigate the DERBI interface, choosing their own areas of interest, to learn more about contaminant sources, health impacts, exposure reduction strategies, and broader policy approaches. This application was developed for use as a smartphone application because of the limited availability of computers among PROTECT participants and the wide availability of smartphones with internet access [17].

### Initial Assessment with PROTECT participants

**Fig 4:**
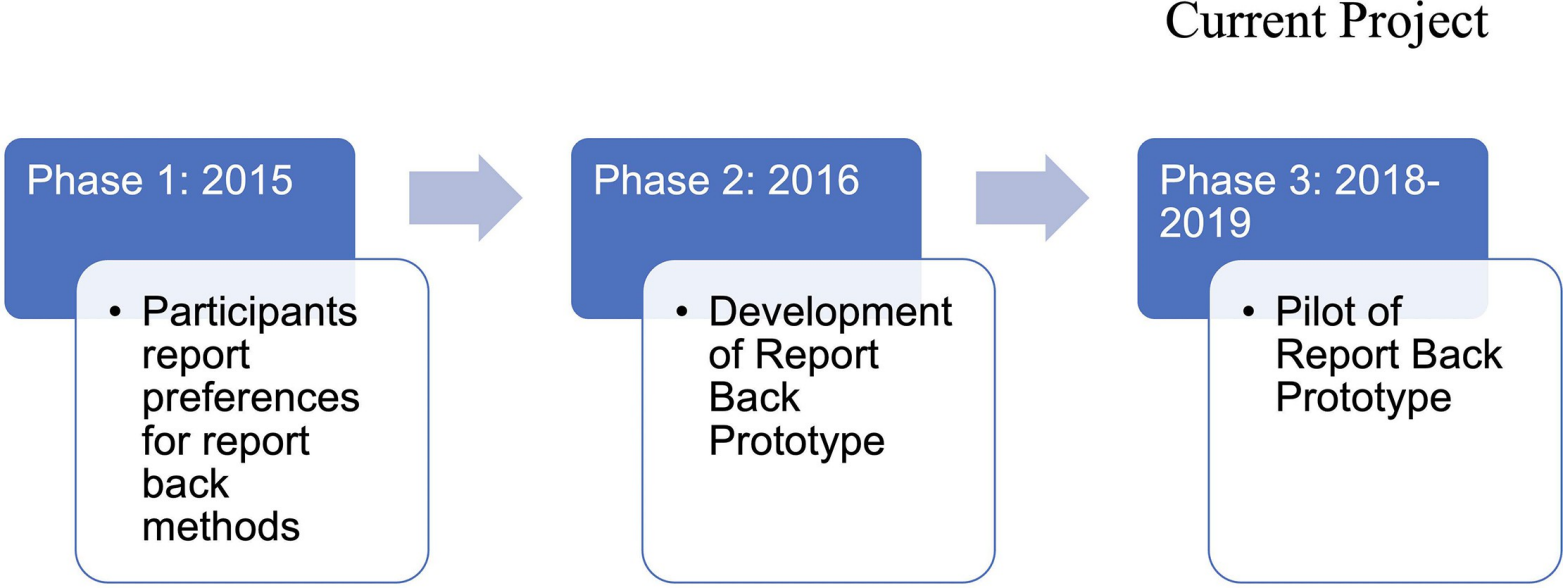
Timeline of Report-Back Process.

To develop the DERBI prototype, we conducted a series of focus groups and key informant interviews with participants and staff from Community Health Centers collaborating with PROTECT, to receive input on knowledge, interest, and preferences about receiving information on the results of their exposure to chemicals [9]. The team leading the focus groups were members of PROTECT’s Community Engagement Core (CEC), which is composed of public health specialists in education, nursing, social work, qualitative methods, and environmental health. Our multisectoral approach also included PROTECT team members from our Call Center, students (Trainees) and data management personnel. The aim of this initial assessment was to learn about the interest and specific preferences participants had on receiving their individual study results (Phase one) to develop the initial smartphone prototype (Phase two). Findings of the first two phases have been shared elsewhere [8–9]. This manuscript focuses on Phase 3. Fig 4 shows the timeline for development of Report-Back methodologies and implementation.

### Recruitment for pilot report-back process

A subset of women was recruited among former PROTECT participants for this report-back pilot test. Recruitment was based on availability of analyzed samples ready to report back. Participants in this group could be from any project year and municipality of the PROTECT Center and could have participated more than once in the project. Participants had previously signed an IRB-approved one-time consent form that included being contacted via PROTECT’s communication center for participation in future projects and agreed to be provided with their personalized results.

### Characteristics of participants

The *Mi PROTECT* prototype was evaluated by 61 participants who attended the in-person report-back meetings. This subset of pregnant women participated in PROTECT between 2011 and 2017. Table 1 describes characteristics of this group and the PROTECT Cohort. Briefly, participants in the report-back group were between the ages of 18–40 years old with a mean age of 27 (SD 6.8), nearly all of them were married or living with a partner (93%) and most had a family income of less $40,000 (60%). In this subset, 5% (n = 3) had recently experienced a preterm birth and 43% (n = 25) reported that their pregnancy in PROTECT was their first child. Additionally, in this subset, one woman was a PROTECT participant for two pregnancies. These characteristics are similar to those observed in the PROTECT Cohort for age, marital status, income, race and pregnancy outcomes.

**Table 1:** Characteristics of PROTECT participants and subgroup of participants in the Report-Back Process.

	PROTECT Cohort		Report Back Subgroup	
	Frequency	Percent	Frequency	Percent
Age	n	%	n	%
Less than 20 years-old	141	9	2	3.4
20 to 24 years-old	444	28.2	6	10.3
25 to 29 years-old	490	31.1	17	29.3
30 to 34 years-old	323	20.5	21	36.2
35 years-old or over	176	11.2	12	20.7
Total	1574	100	58	100
**Hispanic origin**				
No	5	0.3	2	3.4
Yes	1555	99.7	57	96.6
Total	1560	100	59	100
**Race**				
White	774	53.2	27	47.4
Black or African American	39	2.7	5	8.8
More than one race	618	42.5	24	42.1
Other race	14	1.5	1	1.8
Total	1454	100	57	100
**Marital Status**				
Single	291	18.7	3	5.1
Married	822	52.8	48	81.4
Divorced	15	1	Not applicable	
Widow	3	0.2	Not applicable	
Live together	427	27.4	7	11.9
Total	1558	100	58	100
**Income**				
Less than $20,000	650	47.9	16	29.1
$20,000 - $39,999	389	28.6	17	30.9
$40,000 or more	319	23.5	22	40
Total	1358	100	55	100
**Education**				
Less than high school	110	7.1	0	0
High School Degree	422	27.1	9	15.3
Associate’s Degree	345	22.2	8	13.6
Bachelor’s Degree	482	31	30	50.8
Master’s Degree	164	10.5	11	18.6
Doctoral Degree	33	2.1	1	1.7
Total	1556	100		100
**Pregnancy outcomes**				
Full term	1284	78	56	95
Preterm Birth*	125	8	3	5
Early Pregnancy Loss	64	4	Not applicable	
Still birth	18	1	Not applicable	
Pending delivery confirmation	165	9	Not applicable	
Total	1656	100	59	100
**Children before PROTECT**				
No child	683	44.4	25	43.1
One child	610	39.7	19	32.8
Two children	193	12.5	12	20.7
Three or more children	52	3.2	2	3.4
Total	1656	100	58	100

*Preterm birth = <37 weeks of gestational age. Out of 61 participants, 59 provided their PROTECT identification number and were included to describe characteristics of this subset.

### Pilot report-back process

Participants whose samples were ready to report back were invited to receive their personal results in a group meeting. Meetings were held at PROTECT’s research facilities in Manatí and the partner Community Health Centers located in Camuy, Ciales and Morovis in Puerto Rico. Our primary goal was to return results through an innovative approach aiming to enhance social support and to gather feedback to understand if *Mi PROTECT* responded to participants’ interests. During the meetings, analysis of the written content, discussion of the importance of the cultural context and the representation in the images were carried out. At the end of the report-back process, participants were asked to complete two surveys to evaluate the *Mi PROTECT* platform and the report-back training activity. The *Mi PROTECT* evaluation form included eight questions about access and navigation within the platform, adequacy of information and images, assessment of image effectiveness, usefulness and understanding of environmental health information and representativeness of the culture. The report-back training activity survey included questions about relevance of the topic for a better quality of life, motivation to continue learning about the topic, helpfulness of received materials for understanding information presented in the *Mi PROTECT* platform, adequacy of location used, usefulness of *Mi PROTECT* to know more about chemicals that participants are exposed to, interest in receiving a paper copy of results, and knowledge and clarity of presenters of the following topics: Report-Back Process, My Report of Results and Chemical Substances of concern. Both assessments used a Likert-type scale (i.e., strongly agree, agree, disagree, strongly disagree) for each question [18]. Assessments also included an open-ended feedback section at the end. Finally, a private *WhatsApp* group was organized at the request of participants to provide support and exchange information on the *Mi PROTECT* experience. Participants were provided with an educational session that included an interactive presentation, materials in paper format and a water filter that eliminates most of the concerning chemical substances, including lead, which is not a chemical of interest of the study. As an incentive for participation, PROTECT’s CEC provided activities for the participants’ children in a separate room in the PROTECT facility where they were also educated through environmental health-related games and coloring books (https://protect.sites.northeastern.edu/resources/recursos/).

### Statistical analyses

Summary statistics describe PROTECT’s population characteristics from 2011–2019 and the *Mi PROTECT* subsample. Descriptive data that included the frequency and percentage of responses to both the report-back training activity and the *Mi PROTECT* platform (N = 61) were computed. All analyses were conducted using the IBM Statistical Package for Social Sciences Software (SPSS) Statistics for Windows, Version 24.0.

### Ethical approval

The research protocol for PROTECT was approved by the Ethics and Research Committees of the University of Puerto Rico and participating clinics. The Human Research Subjects Protection Office **(**HRSPO) serves as the administrative office for the UPR MSC Institutional Review Boards. Protocol #A8570317. Formal written informed consent was obtained from participants.

## Results

### Report-back activity

Participants provided feedback on how the researchers organized the in-person, group report-back setting. In general, nearly all participants considered that the group report-back process organization was appropriate (n = 59,96%) and characterized the dynamic of the meeting as interesting, n = 58 (95%) (Table 2). The few participants (3% and 4%, respectively) who disagreed with these statements provided suggestions regarding appointment scheduling and meeting organization, which were addressed in subsequent report-back meetings. The vast majority, n = 59 (96%), considered that the report-back process was relevant and will improve their quality of life; about 97% (n = 58) reported that the information received motivated them to continue learning about the topic (i.e., environmental health).

On the Report-Back Process, Chemical Substances and My Report of Results presentations, participants were positively evaluated for their clarity and fluency; 83% (n = 51), 86% (n = 53) and 86% (n = 53), respectively. To achieve success communicating this information, speakers held internal meetings prior to the Report-Back activity and received feedback from PROTECT employees (nurses, health educators, environmental health specialists, and social workers) who are based in Puerto Rico. Presenters of Chemical Substances and Report of Results demonstrated knowledge of the topic from the participants’ point of view (n = 54, 88%). Most participants, n = 53 (87%), cataloged the *Mi PROTECT* platform as useful for learning more about potential chemical exposures, and fewer, n = 42 (71%) also asked for a copy of their results in paper format. Participants also provided suggestions for the training activity, including the need for interactive activities for their children while they (i.e., the moms) received the report-back information.

**Table 2:** Participants in the report-back activity (N = 61).

Regarding the activity, how much do I agree or disagree with:	Strongly disagree		Disagree		Agree		Strongly agree		Total
	n	%	N	%	n	%	n	%	
a) The activity dynamic was interesting.	2	3.3	1	1.6	10	16.4	48	78.7	61
b) The topic of the activity is relevant to improve my quality of life.	2	3.3	0	0.0	5	8.2	54	88.5	61
c) The information received motivates me to continue learning about the subject.	2	3.3	0	0.0	7	11.7	51	85.0	60
d) The presenter of the topic on the Report Back Process expressed themselves clearly and fluently.	2	3.3	0	0.0	8	13.1	51	83.6	61
e) The presenter of the Chemical Substances topic spoke clearly and fluently.	2	3.3	0	0.0	6	9.8	53	86.9	61
f) The presenter of the topic of Chemical Substances demonstrated mastery of the topic.	2	3.3	0	0.0	5	8.2	54	88.5	61
g) The presenter of the topic of My Report of Results expressed themselves clearly and fluently.	2	3.3	0	0.0	6	9.8	53	86.9	61
h) The presenter of the subject of My Report of Results demonstrated mastery of the subject.	2	3.3	0	0.0	5	8.2	54	88.5	61
i) The materials received help me better understand the information presented in My PROTECT Report.	2	3.3	0	0.0	8	13.1	51	83.6	61
j) The facilities were adequate for the activity.	2	3.3	2	3.3	9	14.8	48	78.7	61
k) In general, the organization of this activity was appropriate.	2	3.3	0	0.0	10	16.4	49	80.3	61
l) I understand that the interactive page of My PROTECT Report is useful to learn more about the chemicals to which we are exposed.	2	3.3	0	0.0	6	9.8	53	86.9	61
n) I am interested in a copy of my results on paper.	9	15.3	2	3.4	6	10.2	42	71.2	59

### DERBI report-back platform

Participants provided feedback on the *Mi PROTECT* report-back tool itself. As shown in Table 3, most participants, n = 50 (83%), reported that accessing the platform from mobile phones was “easy” and n = 48 (80%) strongly agreed that it was “easy to navigate.” When evaluating the information provided in the platform, n = 52 (87%) of participants considered that it was relevant, and n = 45 (75%) said that it was easy to understand. Images included in the platform were considered helpful to understanding the information, n = 51 (86%), and n = 57 (96%) reported that the images, examples and language were representative of Puerto Rican culture. In general, most participants, n = 51 (86%), strongly agreed that they will use the PROTECT report-back platform again. Participants also provided recommendations that include: 1) incorporate more avatars and 2) transform the web and mobile platform into a mobile application. Requests from the initial focus group were added to later versions of *Mi PROTECT* to include additional avatars and images that reference the cultural experience and context of the island. Participants requested that multiple avatars should be created that reflected the variety of hair color and types, eye color and skin tones common in Puerto Rico. Another request was that consumer product images should be changed and include currently available products in local Puerto Rican stores.

**Table 3:** Participants in the evaluation of *Mi PROTECT* report-back prototype (N = 61).

Regarding the activity, how much do I agree or disagree with:	Strongly disagree		Disagree		Agree		Strongly agree		Total
	n	%	n	%	n	%	n	%	
a) It is easy to access the platform from my mobile phone.	2	3.3	0	0.0	8	13.3	50	83.3	60
b) The platform is easy to navigate.	2	3.3	0	0.0	10	16.7	48	80.0	60
c) The information on the platform is easy to understand.	2	3.3	0	0.0	13	21.7	45	75.0	60
d) The information included is relevant.	2	3.3	0	0.0	6	10.0	52	86.7	60
e) The images on the platform are adequate.	2	3.3	0	0.0	8	13.3	50	83.3	60
f) The images on the platform help me understand the information.	2	3.4	0	0.0	6	10.2	51	86.4	59
g) The content presented on the platform uses examples, images and / or language representative of our culture.	2	3.4	0	0.0	8	13.6	49	83.1	59
h) I will use the platform again after the activity is finished.	2	3.4	0	0.0	6	10.2	51	86.4	59

## Discussion

To our knowledge, PROTECT is the first research center in Puerto Rico to report back results of individual biological biomarkers and environmental samples provided by participants. This approach offers a democratic, participatory model that will yield improved science, as has been the case with report-back in a variety of environmental health projects [2–3,7]. We would like to highlight the importance of community engagement during the development of the application; the participants’ input was crucial to considering the cultural context and the incorporation of educational information regarding the chemicals as well as recommendations to reduce exposures.

Our results show that the report-back platform was positively received by our participants; most found it easy to navigate and the information useful. In addition, more than half of the participants requested their results in paper format; this may be a focus of future report-back training for PROTECT participants. The report-back training activity, which provided face-to-face interaction and the opportunity for questions and recommendations, was also very well received. This kind of collaboration between researchers and participants can promote environmental health literacy and build trust among research participants. These results draw from the compendium of best practices for reporting back research results to participants, including consulting participants to ensure that the information presented was relevant and culturally appropriate, providing a graphical representation of results in comparison to others in the study, and giving contextual information about contaminants with concrete examples and images. In this way, we have learned to adapt in order to respond to the participants’ objectives and interests by disseminating the findings in a way that they relate to and that makes them feel engaged in the process. One example includes addressing the revisions, changes, and suggestions received, which are shared with our colleagues to be implemented in the *Mi PROTECT* platform. By adapting these practices to a smartphone report, we were able to further enhance the accessibility of the reports and information. Finally, taking into consideration the socio-cultural context of the participants is very important when developing a report-back strategy.

It is important to recognize the strengths and limitations of this approach. We believe this strategy was effective given the active collaboration between researchers and study participants during the development and evaluation of the report-back platform. The participants were not treated as passive subjects, but as individuals with agency whose insights were valued and taken into consideration. Also, digital methods like DERBI reduce practical barriers to report-back and can enable researchers to meet ethical obligations while conducting research, while simultaneously providing participants with the knowledge they need to make informed choices [7,19]. Another advantage of our DERBI *Mi PROTECT* app is the ability of participants to access their data in a secure digital platform along with the ease of returning to the “reducing exposure” section or other sections, as desired. In contrast, printed results reports can appear lengthy and are less able to link to other digital resources [20]. However, recent extreme weather events on the island such as Hurricane Maria [21–22] influence participants’ need for paper reports, which may explain why many participants asked to receive a copy of their results in paper format. This study did not measure the effect of the report-back platform on environmental health literacy (i.e., pre- and post-test), on behavioral changes to reduce exposures (i.e., follow-up surveys), or on actual exposure measurements (i.e., follow-up biological samples). In other words, we do not know if the reporting back of research results has motivated our participants to make changes in their daily lives or has encouraged them to organize as a community to influence local government.

Providing study participants with their results may promote both community engagement and research translation. Many factors could influence the decision of reporting back results in research studies, including the values regarding a participant’s right-to-know, the research team’s perspectives, resources and/or IRB support. According to the Belmont Report principles and community-based participatory research ethics and rights, an informed approach to sharing research results with participants acknowledges their rights and their agency to engage with research findings [7,23]. The 2018 consensus report of the National Academies of Sciences, Engineering, and Medicine elaborates on the ethical principles and benefits to both researchers and study participants [1]. Similarly, in the environmental health context, Brody et al. report that both participants and researchers who participated in report-back initiatives have identified benefits, such as increasing trust in science, better retention in cohort studies, improvement of environmental health literacy, individual and community empowerment, and motivation to reduce household exposures [7]. Our data accords with previous studies that have demonstrated that report-back participants felt respected and recognized their contribution to science [4,19,23–24]. These reports are aligned with the advancement and promotion of environmental health justice and literacy, especially in underrepresented communities.

Information provided through the report-back process helps the PROTECT community plan, design, and cope with challenging circumstances, which include environmental disasters and disparate exposures to harmful chemicals. This bi-directional communication process has been key to designing culturally appropriate changes in newly designed activities, educational strategies and in research overall. Lessons learned from MI PROTECT Report Back were implemented for the development of the “PROTECT Responde” initiative, whose purpose was to create an educational campaign developed for digital social media to inform participants about possible environmental pollutants and harmful chemicals that can be related to adverse effects in maternal and child health. One of the achievements that we have considered a validation of the work is the trust of the participants, particularly those choosing to have more than one pregnancy within the research project, as well as the participation of the general population in our integrated outreach strategies (social media, radio interviews, among others.) Next steps for PROTECT’s CEC/RTC team include developing training for online telecommunication platforms (e.g., Zoom, Teams, Google Meet) to ensure that participants have the option of attending a virtual group report-back meeting online. However, we intend to continue in-person group meetings, with sensitivity to the reality of COVID-19 or other extenuating circumstances of the individual participants.

The *Mi PROTECT* pilot test informs our transdisciplinary research team, community partners and stakeholders of a new way of promoting research right-to-know for study participants. Women who use the *Mi PROTECT* report-back platform considered the information relevant and easy to understand, reflecting PROTECT’s transdisciplinary approaches and its unwavering commitment to our engaged cohort. This study shows how translational science approaches become effective in direct benefit to humans and provides evidence on how participants can have an active role during multiple phases of the research development and process. This study pilots a digital health intervention for underrepresented Hispanic LatinX people, a contribution that targets social and environmental justice.

Finally, it was imperative that our participants feel represented when they access the *Mi PROTECT* platform, and this was achieved given that they considered images, examples, and language representative of their Puerto Rican culture. Researchers and future studies should implement report-back methodologies as an intervention tool to promote fair treatment, environmental health justice and scientific literacy. Researchers should incorporate an evaluation of the report-back process to ensure feedback of participants is considered in future endeavors.
